# Accuracy, concurrent validity, and test–retest reliability of pressure-based insoles for gait measurement in chronic stroke patients

**DOI:** 10.3389/fdgth.2024.1359771

**Published:** 2024-04-03

**Authors:** Saskia Neumann, Christoph M. Bauer, Luca Nastasi, Julia Läderach, Eva Thürlimann, Anne Schwarz, Jeremia P. O. Held, Chris A. Easthope

**Affiliations:** ^1^ DART, Lake Lucerne Institute, Vitznau, Switzerland; ^2^ Cereneo Foundation, Vitznau, Switzerland; ^3^ Therapy Science Group, Lake Lucerne Institute, Vitznau, Switzerland; ^4^ Vascular Neurology and Neurorehabilitation, University of Zurich, Zurich, Switzerland

**Keywords:** stroke, reliability and validity, gait disorders, spatiotemporal gait parameters, wearables, rehabilitation, gait, pressure insoles

## Abstract

**Introduction:**

Wearables are potentially valuable tools for understanding mobility behavior in individuals with neurological disorders and how it changes depending on health status, such as after rehabilitation. However, the accurate detection of gait events, which are crucial for the evaluation of gait performance and quality, is challenging due to highly individual-specific patterns that also vary greatly in movement and speed, especially after stroke. Therefore, the purpose of this study was to assess the accuracy, concurrent validity, and test–retest reliability of a commercially available insole system in the detection of gait events and the calculation of stance duration in individuals with chronic stroke.

**Methods:**

Pressure insole data were collected from 17 individuals with chronic stroke during two measurement blocks, each comprising three 10-min walking tests conducted in a clinical setting. The gait assessments were recorded with a video camera that served as a ground truth, and pressure insoles as an experimental system. We compared the number of gait events and stance durations between systems.

**Results and discussion:**

Over all 3,820 gait events, 90.86% were correctly identified by the insole system. Recall values ranged from 0.994 to 1, with a precision of 1 for all measurements. The F1 score ranged from 0.997 to 1. Excellent absolute agreement (Intraclass correlation coefficient, ICC = 0.874) was observed for the calculation of the stance duration, with a slightly longer stance duration recorded by the insole system (difference of −0.01 s). Bland–Altmann analysis indicated limits of agreement of 0.33 s that were robust to changes in walking speed. This consistency makes the system well-suited for individuals post-stroke. The test–retest reliability between measurement timepoints T1 and T2 was excellent (ICC = 0.928). The mean difference in stance duration between T1 and T2 was 0.03 s. We conclude that the insole system is valid for use in a clinical setting to quantitatively assess continuous walking in individuals with stroke.

## 1 Introduction

With over 12.2 million new cases worldwide, stroke is a significant global health concern and one of the leading causes of disability in the western world (1, 2). Individuals with stroke suffer from motor impairments that substantially affect their gait function (3). In comparison to their healthy counterparts, individuals with stroke frequently display reduced mobility, which negatively influences their participation in everyday activities (4, 5). Therefore, rehabilitation goals commonly focus on enhancing activities such as mobility and participation in daily life (6). One of the main aims of stroke rehabilitation is to achieve each patient's potential for recovery. This includes patient-tailored counseling to maintain and increase physical activity and self-training outside the therapeutic setting (7). This patient-tailored counseling involves a continuous cycle of identification of a patient's trajectory, personalized and precise therapy, and continuous monitoring of progress during the entire patient pathway (8, 9).

Wearables have the potential to serve as valuable tools across the entire patient pathway, by providing assessments of gait and mobility of individuals with stroke both in clinical and home settings. While standardized clinical assessments capture a snapshot of a patient's capacity within a controlled clinical setting, wearables present the opportunity to collect additional information about the quality and quantity of movement, as well as a patient's performance in day-to-day activities (10, 11). This approach provides patients, clinicians, and researchers with the ability to continuously monitor the patient pathway, document progress, and gain insights into the capacity–performance relationship (12). It also assesses whether progress made in rehabilitation can be successfully transferred to daily life (13). A systematic review of the application of wearables in gait rehabilitation of individuals with stroke revealed that accelerometers, activity monitors, and pressure sensors are frequently employed, particularly in hospital-based and inpatient settings. Commonly evaluated gait parameters include gait speed, cadence, step count, and duration of activity (14).

Most metrics for the evaluation of gait performance and quality center around the accurate detection of two main gait events: the foot strike (FS; when the foot first touches the ground), and the foot off (FO; when the foot first leaves the ground) (15). These events, which are well-established and well-described anchors for the calculation of gait quality features, are also relevant for measures of gait quantity, such as the number of steps (16). Therefore, as a first principle, a robust, reliable, and accurate detection of gait events is paramount in a gait measuring system to make it fit for purpose. This has proven challenging over past decades of research, especially in populations with neurological conditions, in which gait patterns are extremely individual-specific and both gait patterns and movement speed are highly variable (17). Compounding this, movement patterns and speeds both change over the course of rehabilitation.

Pressure sensors integrated into insoles are adaptable to various shoe types and contain a standardized arrangement of pressure sensors to ensure both validity and comfort (18). Pressure insoles capture the force interactions between the foot surface area and the ground, presenting an intuitive, straightforward, and reliable method for gait event detection (19). Pressure-based measurement systems such as pressure mats and foot switches have been used as a silver standard for validation and algorithm development studies (20, 21). Various techniques, including piezoresistive, resistive, capacitive, and piezoelectric methods, can be used as mechanisms for sensing plantar force. Different sensor technologies come with distinct sets of advantages and disadvantages. Piezoresistive and resistive methods have a broad force measurement range at a budget-friendly cost but involve the challenges of low repeatability and susceptibility to temperature and humidity. Conversely, capacitive and piezoelectric methods offer increased force sensitivity, but they are susceptible to changes in temperature and interference from electromagnetic sources (22).

Piezoresistive insoles have been validated in a population with neurological conditions aside from stroke, demonstrating their capability to accurately identify 94.5% of all steps, with recall and precision exceeding 0.98 when compared to a Vicon motion capture system (23). In healthy populations, piezoresistive pressure sensors exhibit accuracy levels ranging from an Intraclass correlation coefficient (ICC) of 0.86–0.97, depending on walking speed (24). The FeetMe® Monitor system, validated in individuals with chronic stroke, exhibits an ICC > 0.77 and test–retest reliability of ICC > 0.9 (18). In gait rehabilitation, pressure-based insoles have already been used at different stages of the patient pathway, for example, as a supplementary tool for automatically assessing gait or for identifying activity levels and gait patterns (25–27). David et al. (28) evaluated the rehabilitation progress of 35 individuals in the acute and chronic phase of stroke using 90 s of level walking and the Timed Up and Go Test. Gait parameters, including spatiotemporal (e.g., stride time, step time), kinematic (e.g., angle at FS/FO), and gait cycle events, were assessed using the insole system eSHOE. Before and after four weeks of inpatient rehabilitation, the insoles, which are equipped with a triaxial accelerometer; a triaxial gyroscope; a triaxial magnetometer; and pressure sensors under the big toe, metatarsal heads Ⅰ and Ⅴ, and heel, were used to monitor rehabilitation progress. In a study by Munoz-Organero et al. (29), various walking patterns, including gait asymmetry, heel walking, and a low heel pressure strategy, were assessed in individuals in different rehabilitation stages after stroke during a 10-min walking trial. The insole system consists of eight force-sensitive resistors per insole, which collects data at a frequency of 100 Hz that were further integrated into a personalized self-management rehabilitation system designed for gait relearning. Similarly, a shoe-based sensor system (SmartShoe), which includes five force-sensitive resistors in each flexible insole, has demonstrated acceptable validity for the monitoring of physical activity and common activities of daily life in individuals who are at least three months post-stroke (22).

The major challenge of using wearables for gait measurement in individuals with stroke lies in the precise and consistent measurement of the gait patterns typically associated with hemiparesis. These patterns include decreased stance time on the affected side of the body, longer stance time on the unaffected side, and reduced stride frequency and step length, and all contribute to an asymmetrical walking pattern with fluctuating walking accelerations (30, 31). Despite the widespread use of wearables in research, their application for gait analysis involves limitations in the validity and reliability of spatiotemporal parameters and gait events in individuals with stroke (14).

To address these limitations in validity and reliability of spatiotemporal parameters in individuals with stroke, the purpose of this study was to assess the accuracy, concurrent validity, and test–retest reliability of a commercially available insole system in the detection of gait events and the calculation of stance duration in individuals with chronic stroke. This was accomplished by comparing the data collected from the insoles with a camera-based reference system during continuous walking in a 10-min walking test (10mWT).

## 2 Materials and methods

### 2.1 Participants

Individuals with stroke were recruited from the University Hospital Zurich research database. Inclusion criteria were age ≥ 18 years, chronic stage of stroke (≥ six months since the incident), and ability to walk without assistance on a level surface for at least six minutes (Functional Ambulation Category, FAC ≥ 3). The use of assistive devices was allowed. Potential participants were excluded if they had gait deficits unrelated to stroke or were unable to understand verbal instructions. Seventeen individuals with chronic stroke were recruited for this project. The study was approved by the cantonal ethics committee (BASEC-Nr. Req-2019-00565, Ethics Committee Zurich, Zurich, Switzerland). All participants provided written informed consent after being informed about the study procedure.

### 2.2 Experimental protocol

The participants were invited to attend a single measurement event consisting of two sessions of approximately one hour each. After obtaining informed consent, demographic information and outcome measures were collected to characterize the sample. This included age, gender, time since stroke, type of stroke, use of walking aids, 10mWT, gait speed, six-minute walk test, and Motricity Index. All participants were equipped with insoles placed within their personal footwear while performing two measurement blocks (timepoints T1 and T2), each with three repetitions of the 10mWT. A break of at least 30 min separated the two measurement blocks; during this break, the measurement system was removed from the shoe and charged. Post-break, system mounting was performed from scratch, facilitating the evaluation of test–retest reliability while minimizing the impact of day-to-day variation. All gait tests were recorded with both measurement systems, which were started and synchronized before each test.

### 2.3 Measurement system

A Stappone insole system (version 1.0, stapp one, Vienna, Austria) collected plantar pressure with 12 resistive textile pressure sensors in specific zones under the foot. Additionally, it comprised a triaxial accelerometer, a battery, onboard buffer storage, and a Bluetooth Low Energy 4.2 emitter for each insole. Once the data was collected, parameters were processed onboard the system using a proprietary adaptive thresholding algorithm. The sampling frequency was set to 100 Hz. Raw data for acceleration, plantar pressure, and gait event timings (FS and FO) were recorded and transferred via Bluetooth to the Stappone software (version 0.9.5.6, stapp one, Vienna, Austria) after each trial. The pressure calibration of the insoles was automatically adjusted by the software during postprocessing after each measurement was taken. The insole data were analyzed using an internal threshold-based algorithm for gait event detection. The sensor system was lightweight and could be worn for 36 h before needing a recharge.

**Figure 1:**
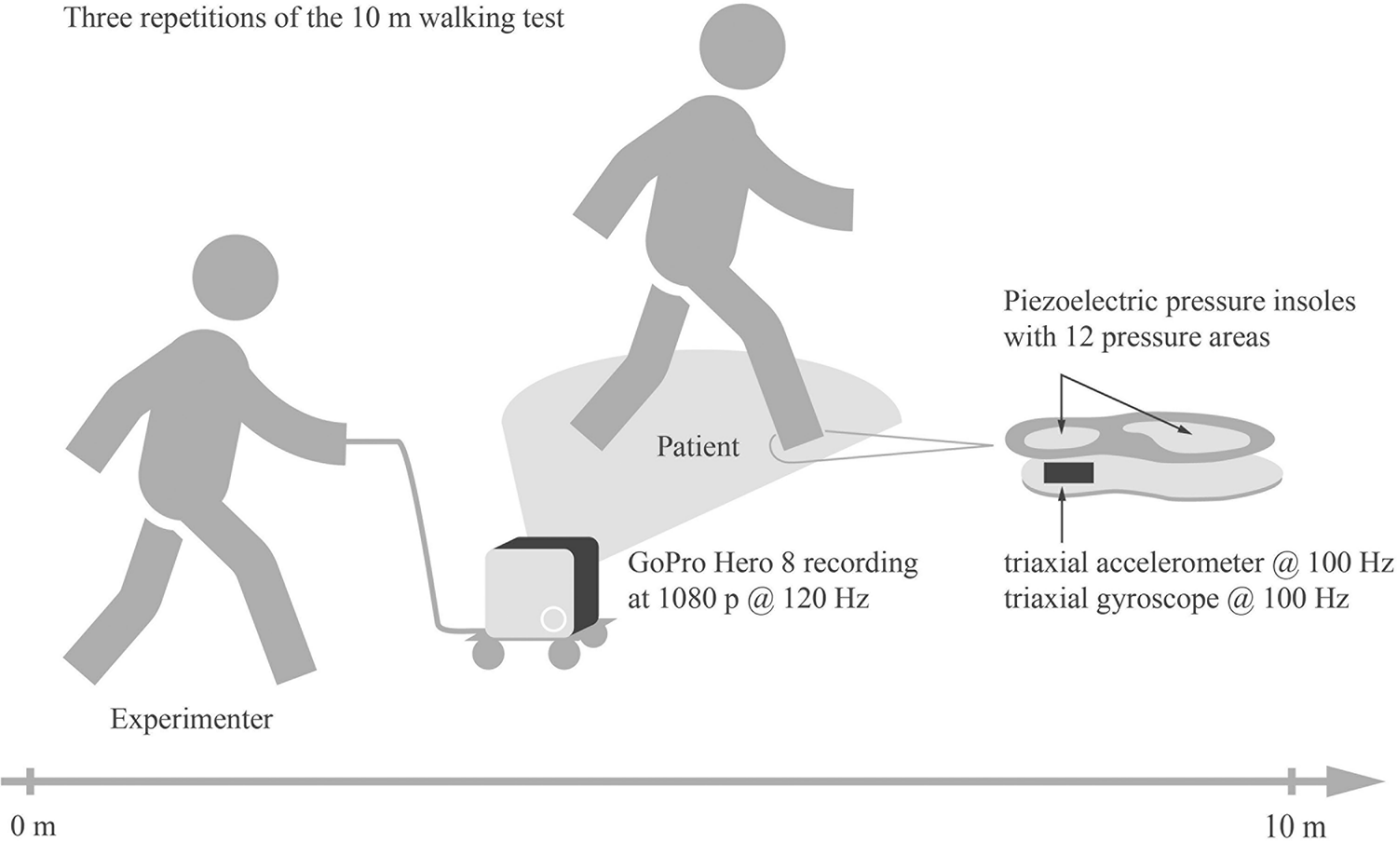
Experimental setup for the 10mWT. Participants completed three repetitions of the 10mWT while wearing the insole system within their personal footwear. As the participants walked, the GoPro camera mounted on the rolling trolley was guided by an assistant walking alongside to capture the gait events during the 10mWT`s.

A GoPro Hero 8 camera (GoPro, Inc., San Mateo CA, USA) served as the reference system because, unlike optoelectronic systems, it does not suffer from the uncertainty associated with human gait labeling (15). The use of manual labeling can lead to inaccuracies due to human error. To minimize these errors, the four-eyes principle was introduced. The camera was securely mounted on a rolling trolley at the height of the feet to capture all trials from a low vantage point with minimal occlusions (Figure 1). The sampling frequency was set to 120 Hz with a resolution of 1,080 pixels and no lens correction. During postprocessing, the gait events (FS and FO) were manually labeled frame by frame by one rater, and the timing was calculated as the time elapsed since the start signal.

Measurement signals were synchronized for each trial using an audio output that was generated by the insoles when the measurement was started. The GoPro was connected to the audio output using a microphone adapter, which recorded the start signal as a sound burst. During postprocessing, this burst was identified with a threshold algorithm and verified manually.

### 2.4 Data preprocessing

The insole system and the reference system were used to assess the stance duration for both the affected and the unaffected side. The accuracy, concurrent validity, and test–retest reliability of stance duration were analyzed from the steps taken in the six 10mWTs per participant. Stance duration, defined as the time between FS and the subsequent FO of the same foot, was calculated for every step. The stance duration was calculated once based on the insole timings and once based on the video reference timings. The timings of FS and FO were calculated as the time elapsed since the starting signal of the video. To ensure correspondence between FS and FO, e.g., that both belonged to the same step on the same side, a plausibility check was performed to remove all out-of-distribution values.

### 2.5 Statistical analysis

The accuracy of the event detection was assessed with the F1 score to balance precision and recall. Precision was defined as the total number of true positive (TP) identifications by the insole system and the reference system divided by the sum of the TPs and the false positives (FPs; events detected by the insole system but not by the reference system).

$$\mathrm{Precision}=\;\frac{\mathrm{TP}}{\left(\mathrm{TP}+\mathrm{FP}\right)}$$

Recall was defined as the number of TPs divided by the sum of the TPs and the false negatives (FNs; events identified by the reference system but not by the insole system).

$$\mathrm{Recall}=\frac{\mathrm{TP}}{\left(\mathrm{TP}+\mathrm{FN}\right)}$$

The F1 score was used to balance precision and recall and was defined as:

$$F1\;\mathrm{score}=2\times \;\frac{\mathrm{Precision}\times \mathrm{Recall}}{\mathrm{Precison}+\mathrm{Recall}}$$

To evaluate the concurrent validity of the insoles, the differences between the stance durations measured by the insole system and by the reference system were analyzed. Normality of the distributions of stance duration was checked by visual inspection of QQ-plots by two investigators. For conflicting ratings, a consensus was reached through discourse. Subsequently, a paired two-sided *t*-test (*α* < 0.05) was employed to detect statistically significant differences between the systems.

Furthermore, the agreement of the stance durations between the two measurement systems was characterized using Bland–Altman plots. Such plots have a strong history in the comparison of reference systems to experimental systems in the medical field (32, 33). Their utility lies in the fact that they not only compare means over the given range of the test population but also enable the identification of fixed and variable bias individually. Both features are important in judging whether a system is sufficiently precise for a given application and population.

Finally, the ICC_2,1_ was used to examine the agreement between the two measurement systems. The ICC_2,1_ was calculated using the following equation:

$$\mathrm{ICC}\;(2,1)=\;\frac{MS_{R}-MS_{E}}{MS_{R}+(k-1)MS_{E}}$$

where MS*_R_* = mean square for rows, MS*_E_* = mean square for error, and *k* = number of raters/measurements (34). According to Fleiss, an ICC ≥ 0.75 is considered “excellent,” 0.40 ≤ ICC < 0.75 is considered “moderate to good,” and ICC < 0.40 is considered “poor” (35). The ICC was applied, as it provides a measure-independent metric of agreement that allows for comparison between different metrics and measurement systems.

In a last comparison, we evaluated test–retest reliability for each system by comparing stance duration data from T1 with data obtained at T2. Differences were identified using a paired two-sided *t*-test after a visual check for normality of the distributions using QQ-plots. The ICC_2,1_ (34) was again applied to permit comparability of reliability to other systems and measures.

## 3 Results

Seventeen individuals with chronic stroke, seven women and ten men, were recruited for the study. The average age was 65.5 years, and the mean time since stroke was 54.7 months. While seven participants were using walking aids outdoors, only five used walking aids during the trials. Descriptive information on the participants’ characteristics is provided in Table 1.

**Table 1:** Characteristics of the study participants (*n* = 17).

Characteristic	Value
Age [years ± SD]	65.5 ± 11.2
Female	7
Time since stroke [months ± SD]	54.7 ± 40.4
Hemiparetic side	
Right Left	6 11
Occasional use of walking aids [%]	41.2
Functional ambulation categories	
4 (independently on even ground) 5 (independently anywhere)	8 9
Gait velocity during 10MWT [m/s ± SD] Test Re-test	0.90 ± 0.37 0.90 ± 0.37 0.92 ± 0.38
Community ambulators (gait speed > 0.8 m/s [%]	58.8
Distance in 6-min walking test [m]	370.4 ± 164.5
Motoricity index (max 100 pts) Ankle dorsal flexion [pts ± SD] Knee extension [pts ± SD] Hip flexion [pts ± SD]	40.3 ± 20.0 24.1 ± 6.6 26.5 ± 3.9 24.6 ± 4.0

Of the 17 participants, one participant was excluded from the analysis due to a measurement system failure. The data from six additional 10mWT trials were not usable due to failure of either synchronization or recording initiation. Additionally, two trials were excluded due to a scrambled gait event sequence proposed by the internal algorithm of the insole system (e.g., multiple consecutive FOs from the same side). Consequently, a total of 88 10mWT trials from 16 participants were included in this analysis. The dataset comprised 3,820 gait events, encompassing 1910 FSs and FOs, covering 995 steps on the right side and 915 steps on the left side.

### 3.1 Gait event detection accuracy

**Figure 2:**
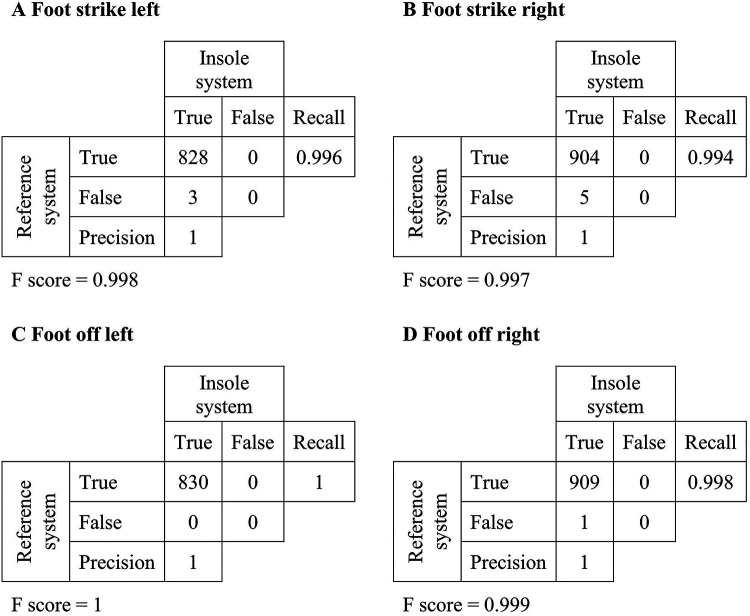
Cumulative confusion matrix for continuous walking. The rows of the table correspond to the total number of gait events detected by the reference system. The columns correspond to the total number of gait events detected during continuous walking by the insole system.

Of all 3,820 gait events, 90.86% were correctly identified by the insole system. A cumulative confusion matrix illustrates gait event detection for continuous walking in all participants by summation (Figure 2). The data analysis showed that 91 right FSs, 86 right FOs, 87 left FSs, and 85 left FOs were not identified as gait events by the insole system. Further investigation showed that all missing events corresponded to either the first FO or the last FS of the trial. To simulate continuous walking, these gait events were excluded from further analysis. Recall values ranged from 0.994 to 1, with a precision of 1 for all measurements. The F1 score ranged from 0.997 to 1, as shown in Figure 2.

### 3.2 Stance duration concurrent validity

Concurrent validity of stance duration was determined through comparison of the insole-derived timings and the video reference measurements. FP events were excluded from the analysis. Sixteen left (1.96% of a total of 815) and 17 right (1.94% of a total of 878) stance durations were excluded as outliers. No systematic reason was identified for the causation of these outliers.

**Figure 3:**
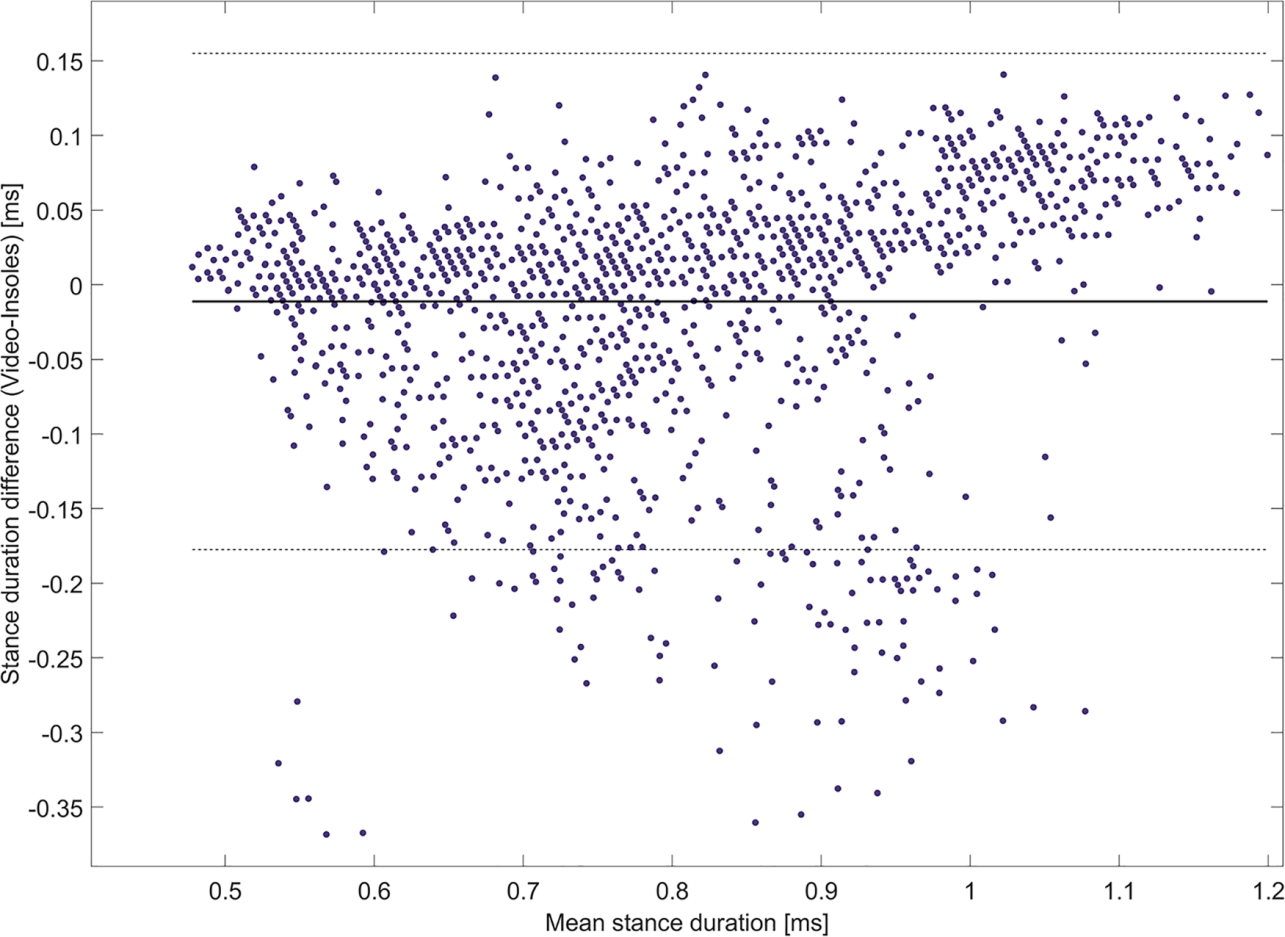
Bland-Altman plot for the comparison of the insole system with the reference system. The mean of the differences is indicated by the solid black line, and 95% limits of agreement are indicated by the dashed lines.

The normality of the distributions of stance duration data was confirmed by both raters using the QQ-plots. The *t*-test performed on the stance durations calculated by the insoles and the reference system revealed no significant difference (*p* = 0.06). The stance durations recorded by the insole system tended to be slightly longer than those recorded by the reference system (difference of −0.01 s). The limits of agreement showed a deviation of insole measurements of up to 0.33 s (Figure 3). The correlation analysis between the insole and the video reference system was robust, with an excellent ICC_2,1_ value of 0.874.

**Figure 4:**
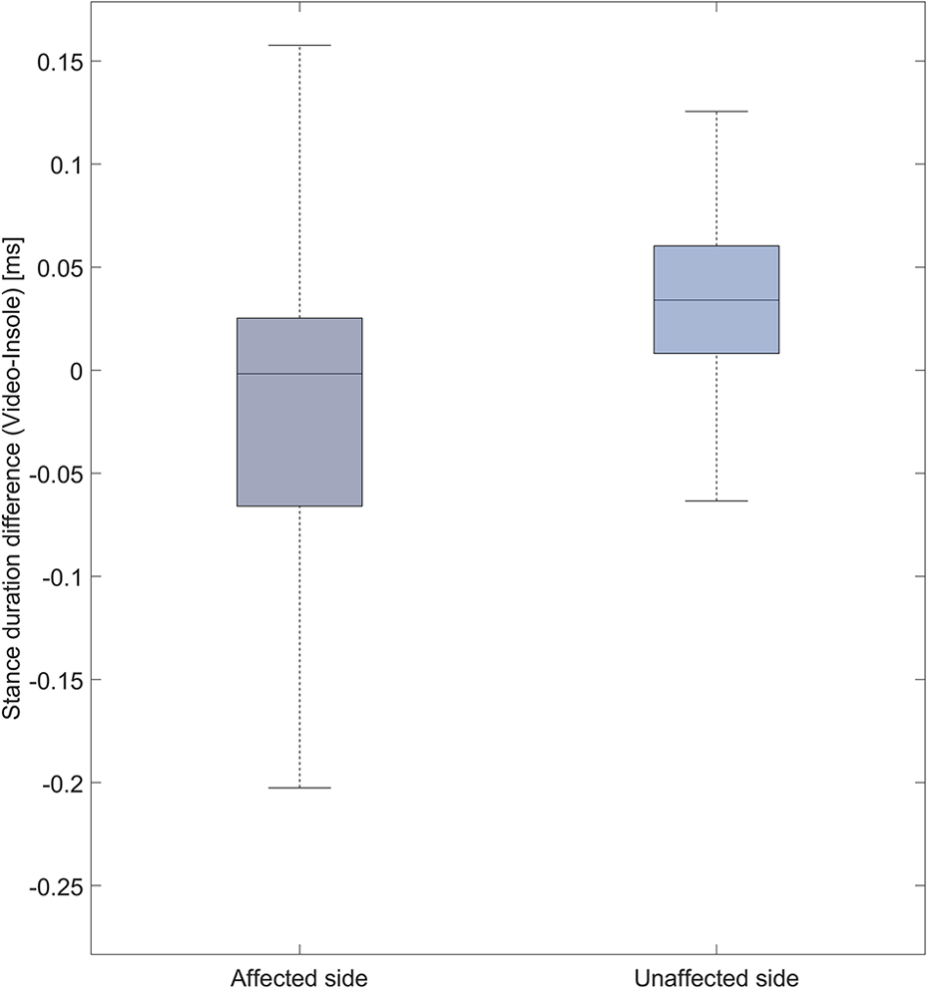
Boxplot representation of the difference in stance duration [ms] between the video system and the insole system for the affected and unaffected side.

**Figure 5:**
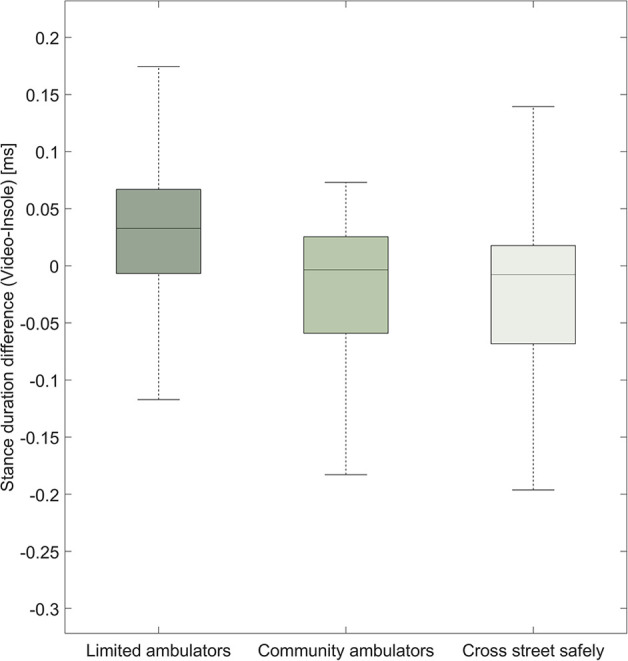
Boxplot representation of the difference in stance duration [ms] between the video system and the insole system for both sides together. We show how the stance duration difference compares between different levels of walking ability.

To enhance the visual clarity of our analysis, we have provided two alternative visualizations of the data reported by the Bland–Altman plot. Specifically, we show the difference in stance duration between both systems categorized by affected vs. unaffected side (Figure 4). Furthermore, we depict differences in stance duration of three categories of ambulators (Figure 5): limited community ambulators (<0.8 m/s), community ambulators (0.8–1.4 m/s), and those able to cross the street safely (>1.4 m/s) (36).

### 3.3 Stance duration test–retest reliability

The normality of the distributions of stance duration data between T1 and T2 was confirmed by both raters using the QQ-plots. The paired, two-sided *t*-test indicated no significant difference (*p* = 0.44) between the two measurement blocks, T1 and T2. The test–retest reliability between measurement timepoints T1 and T2 was excellent, with an ICC_2,1_ of 0.928. The mean difference in stance duration between T1 and T2 was 0.03 s.

## 4 Discussion

The aim of this study was to assess the accuracy, concurrent validity, and -reliability of an insole system in individuals with stroke-related gait deficits during a 10mWT, measured across two sessions in a clinical setting. We targeted a wide functional range of patients to reflect the clinical reality of rehabilitation.

### 4.1 Gait event detection accuracy

The insole system correctly identified 90.86% of the 3,820 recorded gait events. This result aligns with a study that correctly identified 94.5% of all steps in a population with neurological conditions by employing insoles equipped with a piezoresistive device (23). Both recall and precision were >0.98. These rates are comparable to or exceed those reported in studies using accelerometers in individuals with stroke (37, 38). The insole system failed to detect 9.14% of the gait events. Analysis revealed that the initial FOs at the start of the trial and the final FSs at the end of the trial were consistently missed because participants initiated and concluded trials in a standing position. Simulating continuous walking by removing the first FOs and the last FSs from the analysis resulted in a detection error rate of only 0.2%. Among these erroneously detected gait events, eight were FSs detected before the participant started walking, suggesting potential algorithm sensitivity to weight shifts without actual foot movement (39).

### 4.2 Stance duration concurrent validity

The validity of the stance duration measurement was excellent. In comparison to the FeetMe® Monitor system (ICC > 0.77), which was validated in individuals with chronic stroke over three 8-min trials (18), the utilized insole system demonstrated superior performance. Our results align with the accuracy of the ZeroWire® footswitch system, which has four piezoresistive pressure sensors on each foot and was used to record stance time in healthy participants; its accuracy ranged from an ICC of 0.86 to 0.97, depending on walking speed (24). Due to the increased variability and decreased walking speed that are often found in pathological gait, Inertial Measurement Units (IMU) often report reduced accuracy at low walking speeds of 0.4 m/s or severe deviations from normal human gait (40, 41). This is reflected in the lower accuracy of IMU sensors used to detect stance duration in individuals with stroke (42). While we did not record any individuals walking at less than 0.4 m/s, the insole system performed remarkably well for 0.5 m/s–0.6 m/s speeds, which are associated with long stance durations (43). This consistency indicates that the system should function well, even for slow-walking individuals post-stroke.

### 4.3 Stance duration test–retest reliability

The high level of agreement between the two measurement timepoints, T1 and T2, as indicated by the ICC_2,1_ of 0.92, is consistent with findings for the FeetMe® Monitor system (ICC > 0.9) (18). The difference between T1 and T2 can be attributed to typical variation in stance duration (44). These findings indicate that the measures are repeatable over time, even if the insole is removed and reinstalled in the shoe. It is important to note that a short pressure calibration was performed after each introduction of the system into the shoe to compensate for changes in lacing pressure.

### 4.4 Potential use of the insole system in clinical practice

Alterations in walking patterns or the ability to walk can serve as a valuable source of information regarding patient trajectories throughout the rehabilitation pathway and beyond. Gait strategies are influenced by various clinical factors, including neurological, cardiovascular, and musculoskeletal conditions; cardiovascular and metabolic diseases; age-related changes; and trauma (9). Wearables have found extensive application in research, and their utilization is expanding into clinical settings. They can provide information about spatiotemporal, kinematic, and kinetic parameters. Nevertheless, caution is advised in the application of these devices, as they are specifically designed to extract certain parameters in particular patient populations. Therefore, these applications need to undergo a validation process in patients with specific diseases or conditions in clinical settings, as well as in real-world conditions if they are employed in such environments (45). Most importantly, the information gathered using these sensors must align with clinical endpoints and have clinical relevance (46). This ensures that such information can be effectively used by clinicians to provide valuable additional insights into individual health conditions and to facilitate clinical decision-making.

Our findings suggest that the insole system is applicable for assessing stance duration in individuals post-stroke. However, this conclusion is limited to clinical settings and continuous, straight walking. Further research is necessary to determine whether these findings can be transferred to a real-world environment and various activities of daily life.

## 5 Conclusion

In conclusion, the insole system is accurate, valid, and reliable for detecting gait events and calculating stance duration during continuous walking of individuals with stroke, both within and across sessions. Hence, the current model of the insole system can be used in a clinical setting to quantitatively measure gait in such individuals.

## Data Availability

The datasets presented in this article are not readily available due to ethical and legal limitations, as they contain identifiable patient information. Requests to access the datasets should be directed to the corresponding author.

## References

[1] Global Burden of Disease Collaborative Network. Global Burden of Disease Study 2019 (GBD 2019) Healthcare Access and Quality Index 1990–2019. Seattle, WA: Institute for Health Metrics and Evaluation (IHME) (2022). 10.6069/97EM-P280

[2] Rajsic S Gothe H Borba HH Sroczynski G Vujicic J Toell T Economic burden of stroke: a systematic review on post-stroke care. Eur J Health Econ. (2019) 20(1):107–34. 10.1007/s10198-018-0984-0 29909569

[3] Dugan EL Combs-Miller SA. Physiological complexity of gait is decreased in individuals with chronic stroke. Comput Methods Biomech Biomed Engin. (2019) 22(6):658–63. 10.1080/10255842.2019.1578961 30822140

[4] Fulk GD Reynolds C Mondal S Deutsch JE. Predicting home and community walking activity in people with stroke. Arch Phys Med Rehabil. (2010) 91(10):1582–86. 10.1016/j.apmr.2010.07.005 20875518

[5] De Rooij IJM Van De Port IGL Van Der Heijden LLM Meijer J-WG Visser-Meily JMA. Perceived barriers and facilitators for gait-related participation in people after stroke: from a patients’ perspective. Physiother Theory Pract. (2021) 37(12):1337–45. 10.1080/09593985.2019.1698085 31793365

[6] Harris JE Eng JJ. Goal priorities identified through client-centred measurement in individuals with chronic stroke. Physiother Canada. (2004) 56(03):171. 10.2310/6640.2004.00017 PMC3557500 23372280

[7] Morris JH MacGillivray S Mcfarlane S. Interventions to promote long-term participation in physical activity after stroke: a systematic review of the literature. Arch Phys Med Rehabil. (2014) 95(5):956–67. 10.1016/j.apmr.2013.12.016 24389402

[8] Fell N True HH Allen B Harris A Cho J Hu Z Functional measurement post-stroke via mobile application and body-worn sensor technology. mHealth. (2019) 5:47–47. 10.21037/mhealth.2019.08.11 31728382 PMC6851460

[9] Hulleck AA Mohan DM Abdallah N Rich ME Khalaf K. Present and future of gait assessment in clinical practice: towards the application of novel trends and technologies. Front Med Technol. (2022) 4:901331. 10.3389/fmedt.2022.901331 36590154 PMC9800936

[10] Hillel I Gazit E Nieuwboer A Avanzino L Rochester L Cereatti A Is every-day walking in older adults more analogous to dual-task walking or to usual walking? Elucidating the gaps between gait performance in the lab and during 24/7 monitoring. Eur Rev Aging Phys Act. (2019) 16(1):6. 10.1186/s11556-019-0214-5 31073340 PMC6498572

[11] Wu J Kuruvithadam K Schaer A Stoneham R Chatzipirpiridis G Easthope CA An intelligent in-shoe system for gait monitoring and analysis with optimized sampling and real-time visualization capabilities. Sensors. (2021) 21(8):2869. 10.3390/s21082869 33921846 PMC8074136

[12] Pohl J Ryser A Veerbeek JM Verheyden G Vogt JE Luft AR Accuracy of gait and posture classification using movement sensors in individuals with mobility impairment after stroke. Front Physiol. (2022) 13:933987. 10.3389/fphys.2022.933987 36225292 PMC9549863

[13] Maetzler W Rochester L Bhidayasiri R Espay AJ Sánchez-Ferro A Van Uem JMT. Modernizing daily function assessment in Parkinson’s disease using capacity, perception, and performance measures. Mov Disord. (2021) 36(1):76–82. 10.1002/mds.28377 33191498

[14] Peters DM O’Brien ES Kamrud KE Roberts SM Rooney TA Thibodeau KP Utilization of wearable technology to assess gait and mobility post-stroke: a systematic review. J NeuroEngineering Rehabil. (2021) 18(1):67. 10.1186/s12984-021-00863-x PMC8059183 33882948

[15] Wu J Maurenbrecher H Schaer A Becsek B Easthope CA Chatzipirpiridis G Human gait-labeling uncertainty and a hybrid model for gait segmentation. Front Neurosci. (2022) 16:976594. 10.3389/fnins.2022.976594 36570841 PMC9773262

[16] Perry J Burnfield JM. Gait Analysis: Normal and Pathological Function. 2nd ed Thorofare, NJ: SLACK (2010).

[17] Werner C Easthope CA Curt A Demkó L. Towards a mobile gait analysis for patients with a spinal cord injury: a robust algorithm validated for slow walking speeds. Sensors. (2021) 21(21):7381. 10.3390/s21217381 34770686 PMC8587087

[18] Farid L Jacobs D Santos JD Simon O Gracies J-M Hutin E. Feetme® monitor-connected insoles are a valid and reliable alternative for the evaluation of gait speed after stroke. Top Stroke Rehabil. (2021) 28(2):127–34. 10.1080/10749357.2020.1792717 32654627

[19] Crea S Donati M De Rossi S Oddo C Vitiello N. A wireless flexible sensorized insole for gait analysis. Sensors. (2014) 14(1):1073–93. 10.3390/s140101073 24412902 PMC3926603

[20] Aminian K Najafi B Büla C Leyvraz P-F Robert P. Spatio-temporal parameters of gait measured by an ambulatory system using miniature gyroscopes. J Biomech. (2002) 35(5):689–99. 10.1016/S0021-9290(02)00008-8 11955509

[21] Jasiewicz JM Allum JHJ Middleton JW Barriskill A Condie P Purcell B Gait event detection using linear accelerometers or angular velocity transducers in able-bodied and spinal-cord injured individuals. Gait Posture. (2006) 24(4):502–9. 10.1016/j.gaitpost.2005.12.017 16500102

[22] Zhang T Fulk GD Tang W Sazonov ES. Using decision trees to measure activities in people with stroke. 2013 35th Annual International Conference of the IEEE Engineering in Medicine and Biology Society (EMBC). p. 6337–40. Osaka: IEEE, 2013. 10.1109/EMBC.2013.6611003 24111190

[23] Smith BT Coiro DJ Finson R Betz RR McCarthy J. Evaluation of force-sensing resistors for gait event detection to trigger electrical stimulation to improve walking in the child with cerebral palsy. IEEE Trans Neural Syst Rehabil Eng. (2002) 10(1):22–9. 10.1109/TNSRE.2002.1021583 12173736

[24] Pradeau C Sturbois-Nachef N Allart E. Concurrent validity of the ZeroWire® footswitch system for the measurement of temporal gait parameters. Gait Posture. (2020) 82:133–7. 10.1016/j.gaitpost.2020.09.003 32927219

[25] Fulk GD Ryan Edgar S Bierwirth R Hart P Lopez-Meyer P Sazonov E. Identifying activity levels and steps of people with stroke using a novel shoe-based sensor. J Neurol Phys Ther. (2012) 36(2):100–7. 10.1097/NPT.0b013e318256370c 22592067 PMC3355328

[26] Wang C Kim Y Shin H Min SD. Preliminary clinical application of textile insole sensor for hemiparetic gait pattern analysis. Sensors. (2019) 19(18):3950. 10.3390/s19183950 31547437 PMC6767662

[27] Seo M Shin M-J Park TS Park J-H. Clinometric gait analysis using smart insoles in patients with hemiplegia after stroke: pilot study. JMIR Mhealth Uhealth. (2020) 8(9):e22208. 10.2196/22208 32909949 PMC7516684

[28] David V Forjan M Martinek J Kotzian S Jagos H Rafolt D. Evaluating wearable multimodal sensor insoles for motion-pattern measurements in stroke rehabilitation — a pilot study. 2017 International Conference on Rehabilitation Robotics (ICORR). London: IEEE, 2017. p. 1543–48. doi: 10.1109/ICORR.2017.8009467. 10.1109/ICORR.2017.8009467 28814039

[29] Munoz-Organero M Parker J Powell L Mawson S. Assessing walking strategies using insole pressure sensors for stroke survivors. Sensors. (2016) 16(10):1631. 10.3390/s16101631 27706077 PMC5087419

[30] Zhang T Fulk GD Tang W Sazonov ES. Using decision trees to measure activities in people with stroke. 2013 35th Annual International Conference of the IEEE Engineering in Medicine and Biology Society (EMBC). Osaka: IEEE, 2013. p. 6337–40. doi: 10.1109/EMBC.2013.6611003. 10.1109/EMBC.2013.6611003 24111190

[31] Wang Y Mukaino M Ohtsuka K Otaka Y Tanikawa H Matsuda F Gait characteristics of post-stroke hemiparetic patients with different walking speeds. Int J Rehabil Res. (2020) 43(1):69–75. 10.1097/MRR.0000000000000391 31855899 PMC7028468

[32] Giavarina D. Understanding bland altman analysis. Biochem Med (Zagreb). (2015) 25(2):141–51. 10.11613/BM.2015.015 26110027 PMC4470095

[33] Martin Bland J Altman DG. Statistical methods for assessing agreement between two methods of clinical measurement. Lancet. (1986) 327(8476):307–10. 10.1016/S0140-6736(86)90837-8 2868172

[34] Shrout PE Fleiss JL. Intraclass correlations: uses in assessing rater reliability. Psychol Bull. (1979) 86(2):420–8. 10.1037/0033-2909.86.2.420 18839484

[35] Fleiss JL. The Design and Analysis of Clinical Experiments. Nachdr. New York: Wiley (1986. (Wiley series in probability and mathematical statistics Applied probability and statistics).

[36] Middleton A Fritz SL Lusardi M. Walking speed: the functional vital sign. J Aging Phys Act. (2015) 23(2):314–22. 10.1123/japa.2013-0236 24812254 PMC4254896

[37] Macko RF Haeuber E Shaughnessy M Coleman KL Boone DA Smith GV Microprocessor-based ambulatory activity monitoring in stroke patients. Med Sci Sports Exerc. (2002) 34(3):394–99. 10.1097/00005768-200203000-00002 11880800

[38] Saremi K Marehbian J Yan X Regnaux J-P Elashoff R Bussel B Reliability and validity of bilateral thigh and foot accelerometry measures of walking in healthy and hemiparetic subjects. Neurorehabil Neural Repair. (2006) 20(2):297–305. 10.1177/1545968306287171 16679506

[39] Ngueleu A Blanchette A Bouyer L Maltais D McFadyen B Moffet H Design and accuray of an instrumented insole using pressure sensors for step count. Sensors. (2019) 19(5):984. 10.3390/s19050984 30813515 PMC6427154

[40] Chia Bejarano N Ambrosini E Pedrocchi A Ferrigno G Monticone M Ferrante S. A novel adaptive, real-time algorithm to detect gait events from wearable sensors. IEEE Trans Neural Syst Rehabil Eng. (2015) 23(3):413–22. 10.1109/TNSRE.2014.2337914 25069118

[41] Posada-Ordax J Cosin-Matamoros J Losa-Iglesias ME Becerro-de-Bengoa-Vallejo R Esteban-Gonzalo L Martin-Villa C Accuracy and repeatability of spatiotemporal gait parameters measured with an inertial measurement unit. J Clin Med. (2021) 10(9):1804. 10.3390/jcm10091804 33919039 PMC8122546

[42] Lefeber N Degelaen M Truyers C Safin I Beckwee D. Validity and reproducibility of inertial physilog sensors for spatiotemporal gait analysis in patients with stroke. IEEE Trans Neural Syst Rehabil Eng. (2019) 27(9):1865–74. 10.1109/TNSRE.2019.2930751 31352347

[43] Wu AR Simpson CS Van Asseldonk EHF Van Der Kooij H Ijspeert AJ. Mechanics of very slow human walking. Sci Rep. (2019) 9(1):18079. 10.1038/s41598-019-54271-2 31792226 PMC6889403

[44] Hollman JH McDade EM Petersen RC. Normative spatiotemporal gait parameters in older adults. Gait Posture. (2011) 34(1):111–8. 10.1016/j.gaitpost.2011.03.024 21531139 PMC3104090

[45] Salis F Bertuletti S Bonci T Caruso M Scott K Alcock L A multi-sensor wearable system for the assessment of diseased gait in real-world conditions. Front Bioeng Biotechnol. (2023) 11:1143248. 10.3389/fbioe.2023.1143248 37214281 PMC10194657

[46] Brem A-K Kuruppu S De Boer C Muurling M Diaz-Ponce A Gove D Digital endpoints in clinical trials of Alzheimer’s disease and other neurodegenerative diseases: challenges and opportunities. Front Neurol. (2023) 14:1210974. 10.3389/fneur.2023.1210974 37435159 PMC10332162

